# Evaluation of the Safety of Cosmetic Ingredients and Their Skin Compatibility through In Silico and In Vivo Assessments of a Newly Developed Eye Serum

**DOI:** 10.3390/toxics12070451

**Published:** 2024-06-22

**Authors:** Anca Maria Juncan, Luca-Liviu Rus, Claudiu Morgovan, Felicia Loghin

**Affiliations:** 1 Department of Toxicology, Faculty of Pharmacy, “Iuliu Hațieganu” University of Medicine and Pharmacy, 6 Pasteur Str., 400349 Cluj-Napoca, Romania; floghin@umfcluj.ro; 2 SC Aviva Cosmetics SRL, 71A Kövari Str., 400217 Cluj-Napoca, Romania; 3 Preclinic Department, Faculty of Medicine, “Lucian Blaga” University of Sibiu, 2A Lucian Blaga Str., 550169 Sibiu, Romania; liviu.rus@ulbsibiu.ro (L.-L.R.); claudiu.morgovan@ulbsibiu.ro (C.M.)

**Keywords:** cosmetic ingredients, anti-ageing eye serum, safety assessment, skin compatibility, alternative methods, in silico evaluation

## Abstract

The term “risk assessment” is often substituted with “safety assessment”, to demonstrate the safe properties of cosmetic ingredients and formulations. With respect to the actual legislative framework, the proper use of in silico evaluation could offer a representative non-animal substitute for the toxicity evaluation of cosmetic ingredients. The in silico assessment needs to be integrated with other lines of proof (in vitro and/or in vivo data) in the form of a complex methodology in order to demonstrate the safety evaluation of cosmetic ingredients/products. The present study aimed to develop and characterize a new cosmetic formulation, designed for the skin care of the periorbital area. Quality control comprising stability, physicochemical, and microbiological evaluation was performed. Another objective of this study was to present a screening model for the safety evaluation of the cosmetic formulation by identifying individual ingredients, and to confirm the skin compatibility based on in vivo evaluation. The results demonstrated the in silico and in vivo safety profile of the cosmetic ingredients used in the present formulation. In silico evaluation, using a novel, specific software applicable for the risk evaluation of ingredients and formulations, showed that the incorporated ingredients were non-mutagenic and non-sensitizing, and considering the margin of safety (MoS), the cosmetic raw materials could be considered safe. Skin compatibility was confirmed by the patch test performed under dermatological control, evidencing the “non-irritating” potential of the developed cosmetic formulation.

## 1. Introduction

The continuous evolution of the cosmetics industry, together with the development and improvement of the legislation designed to protect the consumer, has contributed to the increasing credibility of cosmetology and cosmetics. Whereas in the past cosmetic products were recommended and mainly used for beauty or hygienic purposes, nowadays they are applied for more complex and demanding objectives, such as maintaining an optimum skin condition, performing a eutrophic function, and finally fulfilling an aesthetic criterion, while being safe and effective. Three main aspects are considered of major importance regarding the formulation and development of cosmetic products—quality, safety, and efficacy [1,2].

According to Regulation (EC) No. 1223/2009 [2], which legally governs cosmetics in the European Union, it must be assumed that cosmetics are safe for human use, and that an assessment of this safety was performed, this assumption being an essential characteristic of the Cosmetics Regulation. Based on this regulation, the consumers are protected against a potential risk (Article 3 regarding safety), by establishing specific rules for the safety assessment of cosmetics (Article 10 referring to safety assessment, to ensure compliance with Article 3) [2,3]. An essential legal requirement is to ensure that the cosmetic formulation has been assessed according to a safety report (Cosmetic Product Safety Report (CPSR), part B) [2].

However, cosmetic formulations contain a mixture of different ingredients with various properties, like emollients, preservatives, humectants, surfactants, fragrances, colorants, and sometimes vegetal extracts [4,5]. Accordingly, even in cosmetics are generally safe for consumers, some allergic effects can be reported [5].

The cosmetics industry is highly motivated to apply non-animal approaches, also known as new approach methodologies (NAMs) or next-generation risk assessment (NGRA), including in vitro, in chemico, and in silico evaluations. These refer to toxicological endpoints and can be applied according to the Cosmetics Regulation No. 1223/2009, which prohibits animal testing, as well marketing of cosmetic ingredients and finished products [6,7].

**Figure 1:**
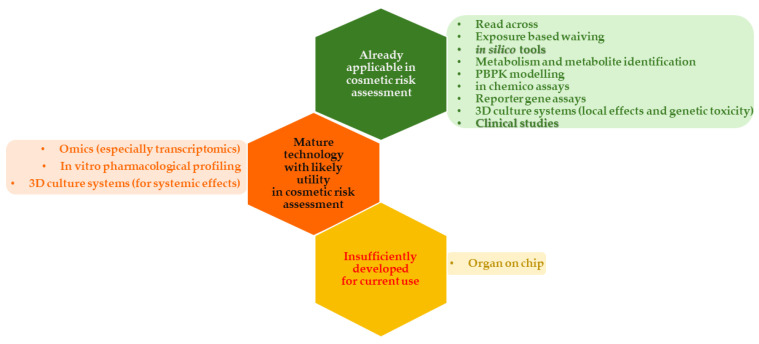
Status of new approach methodologies (NAMs) for the risk assessment of cosmetic ingredients (in silico tools and clinical studies are evidenced as these methods were considered for a comprehensive safety assessment of a novel cosmetic formulation).

NAMs used in the safety evaluation of cosmetic raw materials are nowadays at different stages of implementation, with some already in routine use, while others need more evidence to support their application (Figure 1) [8]. In silico data are useful in regulatory risk assessment when used as supporting evidence in an overall safety evaluation of cosmetic formulations. Figure 1 presents different NAMs, emphasizing in silico methods and clinical evaluation, considered for the safety assessment of a newly developed formulation, as described in this study.

Even if NAMs are currently widely used for the risk assessment of cosmetic ingredients and formulations, there is still a high demand for a combined risk evaluation methodology, as alternative tests are not always fully applicable for multicomponent ingredients and cosmetic formulations, this representing a main disadvantage compared, e.g., to in vivo evaluation [9].

The first objective of the present study was the formulation of an anti-ageing eye serum, specially designed for the periorbital skin area, which incorporates innovative active ingredients such as low-molecular-weight (LMW) HA, claiming an anti-ageing effect, and medium-molecular-weight (MMW) HA, possessing regenerating properties, together with an anti-ageing botanical complex based on horse chestnut flowers. Quality control of the formulation, evaluating the physicochemical characteristics and microbiology, and including a challenge (preservative efficacy evaluation), was performed in this study. Another objective of this study was to carry out an in silico safety assessment, in order to predict the hazards and to evaluate the safety, by characterizing individual cosmetic ingredients of the developed cosmetic formulation. Finally, we sought to confirm the skin compatibility and tolerance through an in vivo assessment.

## 2. Materials and Methods

### 2.1. Selection and Safety-Level Data of Ingredients incorporated into the Cosmetic Formulation

A classification of the raw materials was performed, in order to select the ingredients for the cosmetic formulation (anti-ageing eye serum). Based on material safety data sheets (MSDSs), the following ingredients according to their INCI (International Nomenclature of Cosmetic Ingredients) and commercial denomination were selected for the present formulation:

(i) Butylene glycol cocoate (Cocoate BG, Gattefosse, France) is a multifunctional cosmetic ingredient, which functions as an emollient and solubilizer. It also provides excellent skin compatibility and possesses excellent sensorial properties [10].
(ii) Glycerin (Elton Corporation S.A., Ilfov, Romania) is a safe ingredient, widely used in cosmetics, possessing various functions like serving as a skin conditioning agent, humectant, skin protectant, hair conditioning agent, viscosity-decreasing agent, fragrance ingredient, and denaturant [11,12]. Also, glycerin was demonstrated to have optimal skin tolerability even on atopic dry skin [13].
(iii) As broad-spectrum cosmetic preservatives against bacteria, yeasts, and molds, phenoxyethanol (and) ethylhexylglycerin (Euxyl PE 9010, Schülke&Mayr GmbH, Norderstedt, Germany) were selected [14].
(iv) Low-molecular-weight HA (LMW-HA) (20–50 kDa) (PrimalHyal 50, Givaudan, France) improves skin biomechanical properties, like skin roughness and firmness [15,16], while medium-molecular-weight HA (MMW-HA) (100–300 kDa) (PrimalHyal 300, Givaudan, France) has the capacity to reinforce the skin’s natural defense, along with possessing a regenerating effect [16,17]. Depending on its molecular weight, hyaluronic acid (HA) has different effects in skin care formulations, and in association with other active ingredients, supplementary benefits can be claimed [16].
(v) Fructose (and) glycerin (and) water (and) *Aesculus hippocastanum* (*Horse chestnut*) extract (Gatuline Link n Lift, Gattefosse, France) is a natural complex based on flowers of *Aesculus hippocastanum* (*horse chestnut*), with anti-ageing effects, improving skin texture and especially reducing crow’s feet and under-eye wrinkles (length, surface, and volume) [18,19,20].

Available information on the safe use level and ingredient concentration was retrieved from the Cosmetic Ingredient Database (CosIng) [21] and Cosmetic Ingredient Review (CIR) [22], independent entities responsible for the safety evaluation of individual cosmetic ingredients [23]. Also, COSMILE Europe, a cosmetic ingredient database launched in February 2023 by Cosmetics Europe, was considered in order to find available information on the cosmetic ingredients’ properties, functions, whether they are of synthetic or natural origin, and in which types of formulation they are appropriate [24]. Table 1 presents the general information and safety-level data of cosmetic raw materials in the formulated eye serum according to the CosIng, CIR, and COSMILE databases.

### 2.2. Development and Manufacturing Procedure of the Anti-Ageing Eye Serum

Phase A: Butylene glycol cocoate was heated at 75–80 °C.

Phase B: The aqueous phase, which incorporates ultrapure water (PURELAB^®^ Option Q7 (Type I), ELGA LabWater, High Wycombe, UK), glycerin, and the preservative, was heated to 75 °C. Homogenization was performed to completely disperse the components of the aqueous phase.

Phase A was added to Phase B under continuous stirring, using a T 50 digital ULTRA-TURRAX equipped with a dispersing element S 50 N-G 45 G (IKA, Staufen, Germany) (1600 rpm for 15 min).

Phase C: The active complex fructose, glycerin, water, *Aesculus hippocastanum* (*horse chestnut*) extract, together with LMW and HMW hydrolyzed HA (previously dissolved in 10 mL of water for complete dissolution), were added under constant stirring (600 rpm for 5 min) to the emulsion obtained from phases A and B, previously completely cooled to 40 °C.

### 2.3. Quality Control of the Anti-Ageing Eye Serum

Complying with the requirements of Regulation 1223/2009, various evaluations were conducted for the developed formulation: (a) stability evaluation; (b) physicochemical control: organoleptic testing (appearance, color, odor), pH, density and viscosity evaluation; (c) microbiological control and preservative efficacy test (challenge test) [33,34,35,36,37,38,39].

#### 2.3.1. Stability of the Cosmetic Formulation

For accelerated stability testing, the formulation was alternatively stored for 30 days at 4 °C (16 h) (LKUv 1610 MediLine, Liebherr, Germany), 20 °C (8 h), and 40 °C (16 h) (natural convection drying oven SLN-32 (STD), Pol-Eko, Wodzisław Śląski, Poland) [33,36,37].

#### 2.3.2. Physicochemical Testing of the Developed Formulation

Another criterion for the quality evaluation was the physicochemical control. Thus, organoleptic testing (appearance, color, odor) (ISO 6658:2005 p. 5.4.2 [40]) was carried out and the pH (PB-234 ed. I of 03.10.2013r.), density (20 °C) (PB-155 ed. I of 2 May 2012), and viscosity (Brookfield DV-III Ultra, spindle Sc4-18/RPM: 250 (o/min/shear rate 330 (1/s)) were determined.

#### 2.3.3. Microbiological Quality and Challenge Testing of the Cosmetic Formulation

The microbiological control of the formulation was performed applying standard methods: enumeration and detection of aerobic mesophilic bacteria [41], yeast and mold counts [42], and *Staphylococcus aureus* [43], *Candida albicans* [44], *Escherichia coli* [45], and *Pseudomonas aeruginosa* [46] detection.

The efficacy of the preservation system, respectively, phenoxyethanol and ethylhexylglycerin incorporated in the developed formulation, was also evaluated according to the international cosmetics challenge test standard (PN EN ISO 11930:2012 [47]) [33].

### 2.4. Safety Assessment of the Anti-Ageing Eye Serum

#### 2.4.1. In Silico Approaches for Safety Evaluation of Cosmetic Ingredients and Risk Assessment of the Developed Anti-Ageing Serum

For this purpose, we used a special software, a system dedicated to the specific field of cosmetics, SpheraCosmolife (SpheraCosmolife v. 0.24), which is a module of LIFE VERMEER delivered by Kode Chemoinformatics together with the Istituto di Ricerche Farmacologiche Mario Negri IRCCS, Milan, Italy [48]. The software, applicable for integrated hazard and exposure assessment of cosmetic ingredients and formulations as part of risk evaluation, was implemented for the in silico assessment of the novel developed anti-ageing serum.

The evaluation process is defined within the Scientific Committee on Consumer Safety (SCCS) Notes of Guidance (NoG) for the testing of cosmetic ingredients and their safety evaluation [49]. The margin of safety (MoS), considering the systemic exposure dose, including several models for exposure and risk prediction, and also the threshold of toxicological concern (TTC), are determined with the aid of this software. For a substance to be considered safe, the MoS must be higher than 100, as specified in the SCCS NoG and initially proposed by the World Health Organization (WHO), when defining an interspecies and intraspecies factor each of 10. The software also indicates other toxicological features (e.g., mutagenicity and skin sensitization), to assure an overall assessment of the potential risk of the incorporated cosmetic ingredients [50].

#### 2.4.2. Clinical Safety Evaluation of the Developed Cosmetic Formulation—Dermatological Semi-Open Test

The present study aimed to evaluate the sensitizing/irritant potential and the skin tolerance. After the application of the formulation, under a patch test, the probability of erythema or skin edema appearance was evaluated. This study included 25 healthy Caucasian people, with phototype I-IV according to the Fitzpatrick scale. Subjects with a known history of a dermatological, medical, and/or physical condition that could influence the outcome of this study were not included. Subjects’ skin conditions were considered as general inclusion criteria in this study (skin without irritations and changes requiring pharmacological treatment, subjects using any treatment on the test site, having any active skin disease that could interfere with the purpose of this study). None of the subjects reported previous hypersensitivity or adverse reactions to the individual ingredients of the cosmetic formulation.

The patch (12 mm diameter Finn Chamber, SmartPractice, Phoenix, AZ, USA) was applied (for 48 h) on the arm or interscapular area. A “blank” control sample and a control sample with water were used to avoid inaccurate interpretations related to skin irritations. The skin reaction was examined by a dermatologist 30 min after patch removal. Other evaluations were performed 72 h and 96 h after application.

For clinical safety evaluation, evaluation parameters of skin reactions were assessed by (I) clinical erythema assessment on a six-point severity scale (0 = no erythema; 0.5 = light erythema; 1 = erythema and/or papules; 2 = erythema and/or papules and/or vesicles; 3 = erythema and/or papules and/or vesicles and/or blisters; 4 = erythema, bullous and/or ulcerative reaction and/or papules and/or vesicles and/or blisters), and by (II) clinical edema assessment based on a five-point severity scale (0 = no edema; 1 = very light edema, hardly visible; 2 = light edema; 3 = moderate edema; and 4 = strong edema (extended swelling even beyond the application area)) at all evaluation time points.

The results are expressed based on the average irritation index (X_av_). The formulation could be considered “not-irritating”, “slightly irritating”, “moderately irritating”, or “highly irritating” [33,51,52].

An informed consent form (ICF), including information about the purpose of this study, methodology, and possible side effects was filled in by the volunteer subjects. This study was conducted by an external laboratory (J.S. Hamilton Poland Sp. z o.o., Gdynia, Poland) in accordance with the recommendations of the Cosmetics Regulation and current guidelines [48,49,50].

## 3. Results

### 3.1. Anti-Ageing Eye Serum Formulation

MSDSs of cosmetic ingredient categories incorporated in the formulation were accessed for their INCI denomination, physicochemical characteristics, toxicological evaluation, compatibility with other cosmetic ingredients, and concentration level in cosmetic formulations. Available safe-level information for ingredients and safety information considering use restriction according to the current legal framework were obtained from the CosIng, CIR, and COSMILE databases. Considering all this information, a selection of cosmetic ingredients was performed. Table 2 presents the developed anti-ageing eye serum considering the commercial and INCI denomination of the ingredients, their function in the cosmetic formulation, the supplier, and the concentration limit.

### 3.2. Quality Control of the Anti-Ageing Eye Serum—Physicochemical Characterization and Microbiological Evaluation

Considering the quality characteristics, several evaluations were conducted, in order to demonstrate the physicochemical and pharmacotechnical properties of the developed anti-ageing eye serum. The results demonstrated the stability of the developed formulation under the performed study and conditions, and they showed that it possesses adequate physicochemical properties. Table 3 presents the results of the physicochemical tests of the anti-ageing eye serum, initially and after 30 days, while the formulation was maintained alternatively at different temperatures (4, 20, and 40 °C).

**Figure 2:**
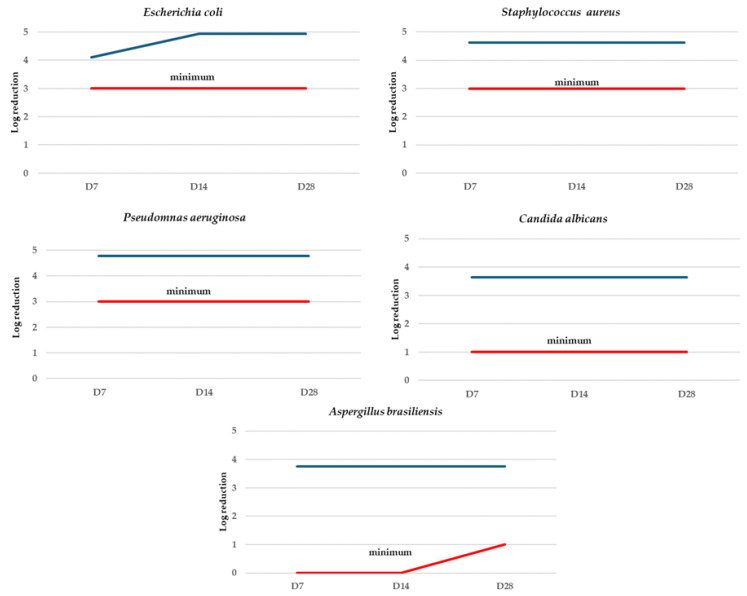
Anti-ageing serum challenge test results. D7—after 7 days; D14—after 14 days; D28—after 28 days. **−−−** antimicrobial protection, **−−−** standard minimum effectiveness.

The microbiological quality of the eye serum was confirmed, and the determinations are presented in Table 4. Also, the challenge test performed evidenced the effectiveness of the antimicrobial protection of the developed cosmetic formulation (Figure 2).

### 3.3. Safety Assessment of the Developed Anti-Ageing Eye Serum

#### 3.3.1. In Silico Assessment for the Safety Evaluation of Cosmetic Ingredients and Risk Assessment of the Formulation

The SpheraCosmolife software provided a summary table (Table 5) of the results for the ingredients incorporated in the eye serum. The results depend on the product type and on the presumed concentrations of the ingredients. The software shows ingredients present in any of the Annexes of the Cosmetics Regulation. Also, it presents the mutagenicity (Ames test), skin sensitization, the dermal absorption according to the Kroes approach, the MoS, and the TTC.

**Figure 3:**
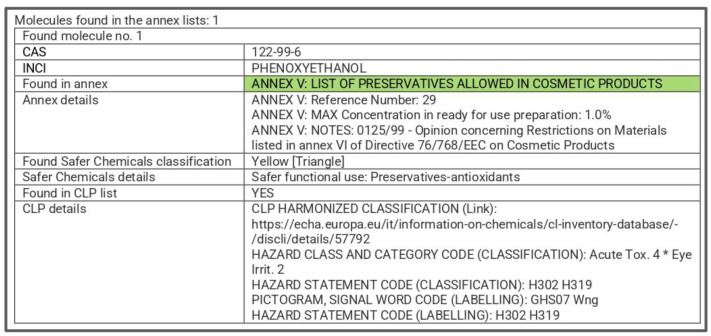
Regulatory information provided by the SpheraCosmolife software for phenoxyethanol (*—Phenoxyethanol could cause serious eye irritation, considering the hazard statement according to ECHA).

For instance, in the developed cosmetic formulation, phenoxyethanol is the only restricted ingredient listed in Annex V of the Cosmetic Regulation (the maximum admissible concentration is 1%). A lower concentration than 1% (in our case 0.9%) must be introduced in the software and used, so that the formulation is safe and complies with the legislation. Moreover, in the “details” section regarding the provided regulatory aspects, the software checked if the ingredient is classified according to the Classification, Labelling and Packaging (CLP) regulation (EC N°1272/2008) (Figure 3).

The safety of phenoxyethanol, which was considered as a preservative complex together with ethylhexylglycerin in the developed formulation, was also supported by the MoS value calculated by the applied software, which in this case was much higher than 100 (MoS = 460.28), meaning it can be concluded that phenoxyethanol is safe at the proposed concentration.

**Figure 4:**
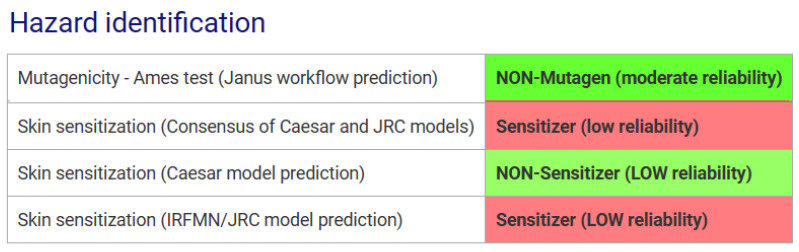
The output of the SpheraCosmolife software for the hazard identification for hydrolyzed HA (0.7% in the anti-ageing eye serum).

The SpheraCosmolife software provided valuable data for mutagenicity and skin sensitization hazard identification and characterization, especially for hydrolyzed HA (LMW- and MMW-HA) at a concentration of 0.7% in a cosmetic formulation specifically recommended for the periorbital area. The software reported values for HA regarding mutagenicity (Ames integrated model) and skin sensitization (Caesar, DT model, and integrated model). Figure 4 presents information on the hazard assessment for the above-mentioned example. The provided values are highlighted with green or light green when they predict the ingredient as safe, while the other values are marked with pale red and predict a low reliability for skin sensitization.

#### 3.3.2. Anti-Ageing Serum Skin Tolerance—Dermatological Semi-Open Test

A very good skin tolerance was demonstrated for the developed cosmetic formulation. According to the patch test performed under dermatological control, none of the subjects included in this study reported any allergic reactions and/or irritation at T_1_ (48 h after product application) or at T_2_ (72 h after product application). Considering nonpositive skin reactions of the subjects at T_1_/T_2_, no further evaluation was performed at T_3_ (96 h after application). The sum of negative reactions (erythema and edema) represents the average irritation index (X_av_), calculated as the average of readings obtained on the subject panel (n = 25). The irritant potential of the tested formulation according to X_av_ was evaluated considering a four-scale classification (X_av_ < 0.50—“not irritating”, 0.50 ≤ X_av_ < 2.00—“slightly irritating”, 2.00 ≤ X_av_ < 5.00—“moderately irritating”, and 5.00 ≤ X_av_—“highly irritating”). Based on this evaluation, it can be concluded that the developed anti-ageing serum meets the requirements of the skin compatibility test and can be described as “not irritating” (X_av_ < 0.50) (Table 6).

## 4. Discussion

The purpose of the Cosmetic Regulation is to protect consumers from potential health hazards and help them make informed decisions when purchasing cosmetic products. According to the present regulation, three main aspects are essential regarding a cosmetic formulation: quality, safety, and efficacy [2,3,38].

Hyaluronic acid represents a popular active ingredient, which is used in skin care products due to its topical benefits recognized as moisturizing, regenerating, or anti-ageing, depending on the molecular weight. Used in combination with other active ingredients, HA or HA derivates incorporated into cosmetic formulations can claim supplementary effects [16]. In the developed formulation presented in this study, LMW-HA and MMW-HA, together with a botanical active complex, were incorporated to sustain the claimed effect of the eye serum. LMW-HA stimulates tight junction protein synthesis, increasing moisturization, and sustains collagen type I synthesis, providing skin firmness. MMW-HA increases fibroblasts and keratinocytes’ proliferation, sustaining the skin cellular regeneration process [15,17,53]. The extract based on Aesculus hippocastanum rich in flavonoids provides periorbital-wrinkle-reducing properties, claiming an anti-ageing effect by sustaining the dermo-epidermal junction integrity, increasing the synthesis of type IV and VII collagen, and holding a smoothing capacity [18,20,54].

Significant data about the ingredients’ safety levels and concentration limits were first accessed through the CosIng, CIR, and COSMILE databases, according to the selection and incorporation of cosmetic ingredients in the developed eye serum, as described in this study.

The quality of a cosmetic preparation is related to the provision of specifications with the expected compliance, such as aspect, color and odor, physicochemical properties, stability, control of contaminants (for, e.g., pathogenic micro-organisms), etc. [55].

In the study performed, a stability test was conducted by exposing samples of the developed cosmetic formulation under accelerated temperature conditions, followed by evaluations of various parameters to observe any physical, chemical, or microbiological changes.

Also, interactions between the cosmetic formulation and the primary packaging should be considered for a complete safety assessment and according to current legislation [38,55].

Safety can be considered the provision of safe products for the general population under normal use conditions, which can be based on three main aspects, namely, ingredient safety, finished product safety, and cosmetovigilance (post-/in-market safety) [3].

The assurance of the safety of a formulation starts from the safe use of ingredients and can comprise two major points: (I) a risk-based assessment of the ingredients, and (II) compliance of the cosmetic product formulation with specific ingredient restrictions according to the current legislative framework [23].

In the European Union, the safety of a cosmetic formulation depends on the safety of the ingredients [56]. Each ingredient incorporated into the cosmetic formulation must prove a toxicological profile [39] broadly described in the MSDS. Generally, the SCCS cosmetic product safety evaluation relies on the principles and practices of the risk assessment process commonly applicable to individual cosmetic ingredients. This risk evaluation procedure is subdivided into four parts: (i) hazard identification, (ii) dose–response assessment, (iii) exposure assessment, and (iv) risk characterization [57,58].

The systemic effects of each ingredient, when evaluating all significant routes of absorption (dermal, oral, or inhalation) and the calculated MoS according to the No Observed Adverse Effect Level (NOAEL), must be considered for the safety profile of a cosmetic ingredient [39]. The SpheraCosmolife software provided data considering the exposure to the individual ingredients, which depended on the inputs in the system, that is, the formulation type and ingredient concentrations. The exposure was calculated for diverse scenarios (based on the parameters defined by the SCCS NoG): (i) absorption of 100% (oral or inhalation), (ii) absorption of 50% (a default value for dermal exposure), or (iii) the more realistic case for dermal absorption based on skin permeation models (the software provides the output of two models for skin permeation, choosing the worst scenario, considering the most conservative of those two values). With the aid of the software, we reported values (experimental or predicted) related to mutagenicity and skin sensitization, and the calculated MoS and the TTC were obtained, assessing the risk associated with the formulation’s ingredients and demonstrating their safe use in the developed anti-ageing eye serum.

A special case of a restricted cosmetic ingredient in the developed formulation that should be taken into consideration is phenoxyethanol, a widely used preservative up to 1% in leave-on and rinse-off cosmetics. It presents high skin penetration, with an existing SCCS opinion confirming the safe use of this substance [27]. However, phenoxyethanol is mentioned in Annex V of the Cosmetics Regulation. Phenoxyethanol is not classified as a skin sensitizer by the European Chemicals Agency, although it showed rare sensitizer properties [59]. When used as a preservative complex with ethylhexylglycerin, phenoxyethanol at a final concentration of 0.9% was demonstrated to be safe according to its toxicity estimation in the developed formulation and in compliance with the legislation.

Skin sensitization, particularly followed by the development of allergic contact dermatitis, is probably the most important adverse reaction to cosmetic ingredients and/or products, considering their dermal route [60,61]. A substantial demand for risk assessment of cosmetic ingredients is to demonstrate that human exposure does not cause skin sensitization [60,62]. Evaluation of skin sensitization was previously performed based on a guinea pig assay (Magnusson and Kligman, Buehler), which was afterwords replaced by a more accurate method, namely the mouse local lymph node assay (LLNA). Nowadays, NAMs are widely applied for the risk evaluation of skin sensitizers [7,60]. In this sense, a very interesting research study was performed and presented by Kalicinska et al., describing an in silico model for the prediction of the sensitizing potential of cosmetic ingredients, especially haptens [63]. According to the values provided by the SpheraCosmolife software, considering the product type and the concentrations used of LMW- and MMW-HA, low skin sensitization was evidenced.

**Figure 5:**
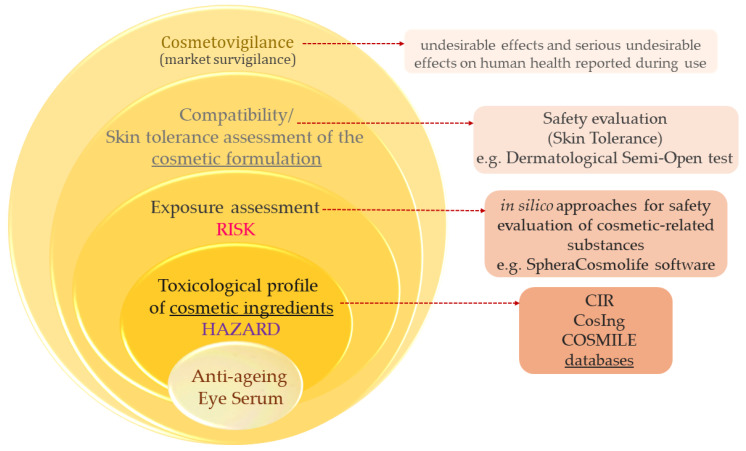
Fundamental aspects of safety assessment of the developed cosmetic formulation.

Finished product safety evaluations ensure that the product is safe, based on the safety profiles of the ingredients, as well as considering the product type and its normal conditions of use and exposure, complemented by a confirmatory safety assessment of the cosmetic formulation. The image in Figure 5 provides an overview of all aspects of the safety assessment for the developed formulation denominated an anti-ageing eye serum, including the special criteria of “ingredients’ safety” and “compatibility of the finished product”, including the “experience from the market” (market surveillance, cosmetovigilance), which should be considered according to the legal framework.

The above-described safety details provide useful information supporting the formulation’s consumer safety. The results of the screening evaluation methodology applied for the assessment of the novel cosmetic formulation confirmed the safety of the individual ingredients and the formulation and the skin compatibility.

Further in vivo efficacy assessment, for, e.g., skin microrelief evaluation and/or periorbital wrinkles’ length and depth reduction, confirmed the cosmetic claim of the anti-ageing formulation, completing our comprehensive characterization and evaluation according to the current legal framework.

## 5. Conclusions

The safety of cosmetic formulations represents an essential and mandatory legislative requirement and also a major industry priority. Cosmetic safety evaluation, implying testing of the ingredients and continuous improvement of the assessment capabilities, is a constant focus of innovation and research activities. The databases that we used review relevant data regarding the safety profiles of the cosmetic ingredients selected for the newly developed anti-ageing eye serum, and it can be stated that they are safe in their current usage. Compliance with the microbiological specifications was confirmed and we found that the physicochemical characteristics were adequate for the developed formulation. With the aid of the SpheraCosmolife software, risk characterization of the cosmetic raw materials used in the developed anti-ageing serum was performed, according to the process defined within the SCCS NoG. The MoS and TTC were calculated, together with the evaluation of other toxicological properties (mutagenicity and skin sensitization), confirming the safety of the cosmetic ingredients used. Phenoxyethanol, incorporated as a preservative in the developed cosmetic formulation, is the only restricted ingredient, listed in Annex V of the Cosmetic Regulation, but when used at a correct concentration level below 1%, it is confirmed to be safe. The SpheraCosmolife software provided valuable data regarding skin sensitization, especially for hydrolyzed HA, which in the proposed concentration of 0.7% and incorporated in a cosmetic product formulated for the periorbital area, showed low sensitizing properties. In vivo evaluation proved the skin compatibility through a lack of a sensitizing/irritant effect under a patch test for the developed cosmetic formulation.

Based on the complexity of these data, a screening safety evaluation method was performed by identifying individual ingredients, together with skin compatibility confirmation through in vivo evaluation. A combined risk assessment approach is still needed, considering that alternative testing methods are not always fully appropriate for multicomponent ingredients/formulations, with this representing the main disadvantage compared to in vivo evaluation.

## Figures and Tables

**Table 1:** General information and safety-level data of incorporated cosmetic ingredients according to the CIR, CosIng, and/or COSMILE databases.

INCI Name	CAS Nr.	Description	Cosmetic Restriction	Maximum Conc. in Ready for Use Preparation	Function(s)	SCCS Opinions	Ingredient Status Database (CosIng */CIR **/COSMILE ***)	Reference
Aqua	7732-18-5	Water	N	N	Solvent	NA	CosIng/COSMILE	NA
Butylene Glycol Cocoate	73138-39-3	Coconut oil fatty acids, 2-hydroxybutyl ester	N	N	Surfactant—emulsifying Emulsion stabilizing	NA	CosIng/CIR/COSMILE	[25]
Glycerin	56-81-5	Glycerol	N	N	Denaturant Hair conditioning	NA	CosIng/CIR/COSMILE	[26]
Phenoxyethanol	122-99-6	2-Phenoxyethanol	Annex V of the EU Cosmetics Regulation (1223/2009/EU)	1%	Antimicrobial Preservative	Opinion concerning restrictions on materials listed in annex VI of Directive 76/768/EEC on Cosmetic Products **** Opinion on phenoxyethanol	CosIng/CIR/COSMILE	[27,28,29]
Ethylhexylglycerin	70445-33-9	3-[2-(Ethylhexyl)oxyl]-1,2-propandiol	N	N	Deodorant Skin conditioning	NA	CosIng/CIR/COSMILE	[30]
Hydrolysed Hyaluronic Acid		Hydrolyzed hyaluronic acid is the hydrolysate of hyaluronic acid derived by an acid, enzyme or another method of hydrolysis	N	N	Hair conditioning Humectant	NA	CosIng/CIR/COSMILE	[31]
Fructose	57-48-7	/	N	N	Humectant	NA	CosIng/CIR/COSMILE	[32]
*Aesculus hippocastanum* (*Horse chestnut*) Extract	8053-39-2	*Aesculus hippocastanum* flower extract is the extract of the flowers of *Horse chestnut*, *Aesculus hippocastanum L.*, Hippocastanaceae	N	N	Skin conditioning	NA	CosIng/COSMILE	NA

* CosIng—Cosmetic Ingredient Database, ** CIR—Cosmetic Ingredient Review, *** COSMILE Europe—Cosmetics Europe database; SCCS—Scientific Committee on Consumer Safety; **** the previous Directive 76/768/EC (adopted on 27 July 1976) was replaced by the EU Cosmetics Regulation (1223/2009/EU), adopted in 2009 and fully in force since July 2013; N—not applicable; NA—not available.

**Table 2:** The anti-ageing eye serum formulation.

Commercial Name	INCI	Function	Supplier	INCI-KEY * (%)
Aqua	Water	Solvent		A
Cocoate BG	Butylene Glycol Cocoate	Emollient/solubilizer	Gattefossé	E
Glycerol	Glycerin	Denaturant/humectant/ solvent	ELTON	E
Euxyl PE 9010	Phenoxyethanol and Ethylhexylglycerin	Preservative	Schülke & Mayr GmbH	F
PrimalHyal™ 50	Hydrolyzed Hyaluronic Acid	Antistatic/humectant/ skin conditioning /moisturizing	Givaudan Active Beauty	F
PrimalHyal™ 300	Hydrolyzed Hyaluronic Acid	Antistatic/humectant/ skin conditioning/moisturizing	Givaudan Active Beauty	F
Gatuline Link n Lift	Fructose (and) Glycerin (and) Water (and) *Aesculus hippocastanum* (*Horse chestnut*) Extract	Active ingredient/ anti-ageing	Gattefossé	E

* INCI Key A > 50%; 1% < E ≤ 5%; 0.1% < F < 1%.

**Table 3:** Anti-ageing eye serum physicochemical properties.

Parameter	Unit	Results	
		Initial	After 30 Days
Viscosity at 20 °C (Brookfield DV-III Ultra)	mPa·s	6.43 ± 0.07	7.47 ± 0.08
Density at 20 °C (PB-155 ed. I of 02.05.2012)	g/cm^3^	1.016 ± 0.003	1.015 ± 0.003
Organoleptic testing (ISO 6658:2005 p. 5.4.2)			
Appearance		W/O mixture *	Liquid **
Color		Beige	Light yellow
Odor		Specific of cosmetic ingredients	Characteristic of ingredients
Consistency		Liquid	Liquid
pH (PB-234 ed. I of 03.10.2013r.)		5.5 ± 0.2	5.6 ± 0.2

* W/O—water in oil; **—without mechanical impurities.

**Table 4:** Microbiological evaluation of the developed anti-ageing serum.

Parameter	Standard	Result (CFU/g)	Admissibility Limit (CFU/g)	Concordance
Enumeration and detection of aerobic mesophilic bacteria	ISO 21149:2017 [41]	<10	<100	**√**
Yeast and mold count	ISO 16212:2017 [42]	<10	<10	**√**
*Staphylococcus* *aureus* detection	ISO 22718:2016 [43]	-	-	**√**
*Candida albicans* detection	ISO 18416:2016 [44]	-	-	**√**
*Escherichia coli* detection	ISO 21150:2016 [45]	-	-	**√**
*Pseudomonas* *aeruginosa* detection	ISO 22717:2016 [46]	-	-	**√**

“-”—absent; CFU—colony-forming units.

**Table 5:** Hazard and exposure specifications of the cosmetic ingredients incorporated into the anti-ageing eye serum.

Ingredient ID	CAS	INCI	Conc. % (*w*/*w*)	Annex	Mutagenicity	Skin Sensitization	Dermal Abs.	MoS	TTC *
Deionized Water	7732-18-5	Aqua	92.55	-	-	-	-	-	-
Cocoate BG	73138-39-3	Butylene Glycol Cocoate	1.00	-	-	-	-	-	-
Glycerin	56-81-5	Glycerin	4.00	-	NON-Mutagen	NON-Sensitizer	80%	334.07	**0.046 mg/kg bw/day**
Phenoxyethanol	122-99-6	Phenoxyethanol	0.90	V	NON-Mutagen	NON-Sensitizer	80%	460.28	**0.046 mg/kg bw/day**
Ethylhexylglycerin	70445-33-9	Ethylhexylglycerin	0.10	-	NON-Mutagen	NON-Sensitizer	80%	9737.47	**0.0023 mg/kg bw/day**
Hydrolyzed Hyaluronic Acid	9004-61-9	Hydrolyzed Hyaluronic Acid	0.70	-	NON-Mutagen	Sensitizer	10%	9,713,408.1	**0.0023 mg/kg bw/day**
Fructose	57-48-7	Fructose	2.50	-	NON-Mutagen	NON-Sensitizer	80%	2120.28	**0.046 mg/kg bw/day**
*Aesculus hippocastanum* (*Horse Chestnut*) Extract	8053-39-2	*Aesculus hippocastanum* (*Horse Chestnut*) Extract	0.50	-	-	-	-	-	-
	experimental value		good reliability		moderate reliability		low reliability NON-Senzitizer		low reliability Senzitizer

Product type: **anti-ageing eye serum**. *—TTC values according to Cramer class classification and adapted for cosmetic ingredients, as calculated by SpheraCosmolife: Class I—low toxicity (0.046 mg/kgbw/d) and Class II—medium toxicity (0.023 mg/kgbw/d).

**Table 6:** Skin responses of the 25 subjects included in the semi-open test for the assessment of the irritating and sensitizing effects of the developed anti-ageing eye serum, expressed as the average irritation index (X_av_).

	T_1_ (48 h after Application)	T_2_ (72 h after Application)	T_3_ (96 h after Application)
**Erythema**	0	0	Examination ignored
**Edema**	0	0	Examination ignored
**X_av_**	0	0	

0 values in columns T_1_ and T_2_ refer to the sum of negative reactions (n = 25). X_av_—represents the average irritation index (sum of negative reactions (erythema and edema)).

## Data Availability

Data are contained within the article.
